# Weak Eyes

**Published:** 1856-09

**Authors:** 


### WEAK EYES.

SOME persons are unable to read much, because there is a constant effort to clear away something by winking the eyes, at other times they water, and thus interfere with their useful employment. Under such circumstances, do not hurry off to an Oculist, nor go to poulticing your eyes, nor use any of the hundred and one cures, which reckless and presumptuous ignorance will advise with wonderful volubility and confidence. In many instances, the difficulty may be controlled by darkening the room, letting only a small amount of light fall upon the page or sewing, just enough to enable you to see distinctly without straining. Let the light come in rather from behind, and to one side.

The habit of reading and sewing by artificial light is ruinous to many eyes, and those who persist in it will bitterly regret it in after years.

